# The impact of a group based, remotely delivered weight loss intervention in women with polycystic ovary syndrome on ovulation, quality of life and body composition

**DOI:** 10.3389/frph.2022.940945

**Published:** 2022-07-22

**Authors:** Anna M. Gorczyca, Felicia L. Steger, Lauren T. Ptomey, Robert N. Montgomery, Riley Mickelsen, Patricia Smith, Joseph E. Donnelly, Courtney A. Marsh

**Affiliations:** ^1^Division of Physical Activity and Weight Management, Department of Internal Medicine, University of Kansas Medical Center, Kansas City, MO, United States; ^2^Division of Endocrinology, Diabetes and Clinical Pharmacology, Department of Internal Medicine, University of Kansas Medical Center, Kansas City, MO, United States; ^3^Department of Biostatistics, University of Kansas Medical Center, Kansas City, MO, United States; ^4^Department of Obstetrics and Gynecology, Center for Advanced Reproductive Medicine, University of Kansas Medical Center, Kansas City, MO, United States

**Keywords:** polycystic ovary syndrome, obesity, weight loss, ovulation, quality of life

## Abstract

**Background:**

Obesity and visceral adiposity are associated with anovulation. The most common cause of anovulatory infertility in women of reproductive age is polycystic ovary syndrome (PCOS). We conducted this formative study to examine the effects of a remotely delivered, group-based lifestyle program for women with overweight/obesity and PCOS on ovulation, PCOS related quality of life (PCOSQ) and body composition.

**Methods:**

Women with anovulatory infertility caused by PCOS (*N* = 12) were enrolled in a 6-month high-intensity weight management intervention. Participants were asked to attend 45 min., group behavioral lifestyle sessions, delivered remotely by a registered dietitian weekly across the 6-mo. study and comply with a reduced energy diet, increased physical activity (225 min/wk.), and self-monitoring of weight, physical activity and diet. Diets consisted of five portion-controlled meals (three shakes + two entrees), at least five servings of fruits/vegetables, and ad libitum non-caloric beverages daily. Wilcoxon signed-rank tests were used to assess changes in outcomes across the intervention.

**Results:**

Twelve women received the weight loss intervention (mean age = 32.7 ± 4.2 yrs., BMI = 36.8 ± 4.5 kg/m^2^, 92% college educated), and 8 completed the intervention. Eight (67%) women reported ovulating during the intervention with an average time to ovulation of 57 ± 45 days. Women lost an average of 3.85 ± 5.94 kg (*p* = 0.02), decreased their BMI (−1.61 ± 1.09 kg/m^2^; *p* = 0.04), and waist circumference (−4.54 ± 3.03 cm; *p* = 0.04) over the 6-mo. intervention. Additionally, self-reported menstrual problems measured by PCOSQ significantly improved over the study (*p* = 0.03).

**Conclusion:**

A multicomponent group-based, remotely delivered, lifestyle intervention delivered remotely is a feasible and potentially scalable option to achieve clinically relevant (>3%) weight loss in women with PCOS.

**Clinical trial registration:**

www.clinicaltrials.gov, identifier: NCT03677362.

## Introduction

Polycystic ovary syndrome (PCOS) is the most common endocrine disorder in women of reproductive age characterized by ovarian dysfunction, hyperandrogenism and polycystic ovarian morphology (1, 2). Women with PCOS often have severe metabolic consequences including increased abdominal fat, insulin resistance and metabolic syndrome (3, 4). Due to a potential bidirectional relationship with obesity and PCOS (5), the prevalence of overweight and obesity is significantly higher in women with PCOS compared with women without PCOS, with estimates ranging from 40 to 80% (6). Obesity further heightens the reproductive, metabolic, and psychological dysfunction caused by PCOS (7), and women with both PCOS and obesity are more likely to suffer from anovulation compared to women with PCOS and a normal body weight (8). Lifestyle intervention targeting at least 5–15% weight loss is the recommended first line of treatment for women with PCOS and overweight or obesity (5, 9–11), though there is no established weight or weight loss threshold to induce ovulation or resumption of menstruation in women with PCOS and overweight/obesity.

There is a complex interplay of physical and psychological symptoms in women with PCOS and obesity leading to high rates of depression, stress, anxiety, low self-esteem, low quality of life, and poor body image (12, 13). However, diet and exercise programs can result in weight loss, improved endocrine function, improved quality of life and wellbeing, improved nutrition, and increased pregnancy rates (5, 7, 14–18). Lifestyle interventions in infertile women without PCOS may restore ovulatory cycles, though the impact on live birth rates is inconclusive (19, 20). Still, resumption of menstruation and ovulation is an important starting point to treat infertility and 42–90% of women may resume ovulation during or following a diet and lifestyle intervention (15, 16, 19, 21, 22). Costs associated with a live birth in anovulatory women with obesity are 100% greater than their normal weight counterparts (23). First line medical therapy for anovulation is an ovulatory stimulant such as clomiphene citrate which has a comparable out-of-pocket cost to weight loss treatment over 6 months and results in similar rates of live births (24). However, reports indicate this medication causes depressed mood and/or mood swings in 41 and 45% of women, respectively (25). In contrast, it is well established that lifestyle change programs result in improvements in mood and quality of life (26).

The 2018 International Evidence-Based Guideline for Assessment and Management of Polycystic Ovary Syndrome recommends that a healthy lifestyle and prevention and management of excess weight should be prioritized in all people with PCOS (10). However, the management of PCOS is inconsistent and the needs of these women are not being adequately met (10). Barriers to implementation of lifestyle and/or weight management in those with PCOS are similar to those of the general population as the primary healthcare professionals tasked with treatment of PCOS are not well equipped to deliver lifestyle interventions (27). Insufficient utilization of allied health professionals, such as dietitians, exacerbates dissatisfaction in medical care for these women (27). Improving the availability and cost of programs or professionals to support lifestyle management is key to improving referrals from general practitioners and therefore improve health care for women with PCOS (27). In addition, women with PCOS suffer greater feelings of isolation compared to women without PCOS and may benefit from social support (28) and specifically treatment in a group setting with psychological support (29, 30). Further, increased attention has been given to the need for accessible lifestyle modification programs that decrease common barriers such as time and work commitments, while still producing beneficial psychological and physiological outcomes that are inexpensive for women with PCOS (10). Alternative delivery strategies may be especially important in women with PCOS as younger populations tend to have lower adherence, decreased retention and lower weight loss in lifestyle interventions (31). Thus, replicable, remotely delivered lifestyle change programs can fill a notable treatment gap for this population. Therefore, we conducted a single-arm formative study to examine the effects of a 6-month remotely delivered, multicomponent lifestyle group-based program tailored specifically for women with anovulatory infertility caused by PCOS on ovulation, quality of life and body composition.

## Materials and methods

### Overview

We delivered health education to women with PCOS to improve diet, physical activity, and behavior in a 6-month intervention. We instructed participants to use portion-controlled meals (PCMs), increase fruits and vegetables, increase moderate to vigorous physical activity (MVPA), and to self-report these behaviors weekly. Ovulation was tracked using ovulation prediction kits (OPKs; Pregmate, Fort Lauderdale, FL) and an OvuSense OvuCore™ fertility sensor (Fertility Focus Inc., Old Saybrook, CT). A registered dietitian led group behavioral counseling sessions remotely *via* video conferencing (Skype™, Palo Alto, CA). This study was conducted in the Kansas City Metropolitan Area and was approved by the Institutional Review Board at the University of Kansas Medical Center. The study was registered at clinicaltrials.gov (NCT03677362).

#### Participant eligibility

Participants satisfying the following criteria were eligible for this study. Inclusion: Age 21–42 years, BMI 25–44.9 kg/m^2^, diagnoses of anovulatory infertility caused by PCOS as assessed by Rotterdam criteria (2), verified by their physician, willing to delay fertility treatment for the 6-month intervention and weight stable (±4.6 kg) for the previous 3 months. Women taking metformin were included in the study. Exclusion: A history of another infertility diagnosis other than ovulatory dysfunction resulting from PCOS, unable to participate in moderate vigorous physical activity (such as brisk walking), currently participating in greater than three, 30-min bouts of planned physical activity per week, participating in a weight loss or physical activity program in the previous 6 months, taking a weight loss medication in the previous 2 months, evidence of binge eating disorder, or currently on birth control medication which would prevent ovulation from occurring. Women who became pregnant during the intervention were removed from the study.

#### Recruitment

Participants were recruited from September 2018 to September 2019 through reproductive endocrinology clinics, social media, University of Kansas Medical Center's Healthcare Enterprise Repository for Ontological Narration (HERON), an i2b2-based clinical integrated data repository (32), obstetrics and gynecology clinics, and PCOS support groups. All participants provided written informed consent before engaging in the study.

### Study intervention

Participants completed a 6-month, group-based, weekly, and remotely delivered lifestyle intervention that recommended a reduced-energy diet and increased exercise and delivered behavioral change techniques to facilitate adherence to recommendations.

#### Diet

Guidance to follow a ~1,200–1,500 kcal/day diet as recommended by 2018 International Evidence-Based Guideline for Assessment and Management of Polycystic Ovary Syndrome (10) was provided. A generally healthy, nutrient-balanced, low-fat diet was chosen based on our experience implementing this dietary pattern and current evidence indicating that no specific energy-equivalent diet is better than another in this population (10). Participants were able to choose a combination of two commercially available PCMs, three low-calorie shakes, and at least five one-cup equivalent servings of fruits and vegetables per day. We provided directions and a list of examples to purchase PCMs that contained ~200–300 kcals and had ≤3 g saturated fat. We provided meal replacement shakes (Profile by Sanford Health, Sioux Falls, SD). Participants were able to order from a variety of shake flavors, and the shakes contained ~100 kcal and 15 g protein each.

#### Physical activity

A recommendation to achieve ≥ 225 min/week of moderate-vigorous physical activity (MVPA) was provided as recommended in the “2009 ACSM Position Stand on Physical Activity Interventions for Weight Loss and Prevention of Weight Regain in Adults” (33). This recommendation is line with the 2018 International Evidence-Based Guideline for Assessment and Management of Polycystic Ovary Syndrome to achieve ≥ 150 min/week of MVPA for health and prevention of weight gain, or ≥250 min/week of MVPA for weight loss. The recommendation progressed from 20 min/day-3 days/wk. the first week to 45 min/day-5 days/wk. by week 6 and remained at that level through 6 months. For physical activity to count toward this weekly number, the physical activity had to be aerobic in nature and last ≥10 min in duration. While not counting toward their weekly minutes, participants were also encouraged to include muscle strengthening exercises at least 2 days per week. Participants were asked to increase their daily steps by 10% each week from their current level until reaching a goal of 10,000 steps/day to reduce sedentary activity. Pedometers (Omron HJ-320, Lake Forest, IL) were provided to participants as both a motivational tool and to self-monitor daily activity and were worn over the non-dominant hip.

#### Behavioral intervention

Participants were given a comprehensive program notebook which included general guidelines regarding participation in the study, calendars and timelines for class meetings and data reporting, lessons for each session, and detailed instructions for the diet plan, including appropriate recipes, handouts, worksheets, and assignments specific to the topic for each session. Registered dietitians delivered behavioral counseling sessions *via* group video conferencing (Skype) weekly for 24 weeks. These ~45 min. group meetings utilized behavioral strategies based on Social Cognitive theory (SCT) to promote beneficial lifestyle changes in diet and exercise (34). Counseling strategies included goal setting, self-monitoring, promotion of self-efficacy, manipulation of the environment to promote behavioral change, and reflection on outcome expectations and outcome values. Sessions began with a check-in question designed to identify barriers to diet and exercise to allow the group to work together to identify solutions and to promote group cohesion and social support. Each session included education on a weight management topic, such as reading nutrition labels, planning for social situations, exercising while on vacation, etc. Additionally, PCOS-specific education material was provided, such as the role of nutrition and physical activity on insulin resistance. We provided weekly homework assignments to increase self-efficacy for diet, exercise, and self-monitoring and to encouraging practicing behavioral strategies. Lastly, participants reported their self-monitoring data (diet, PA and weight) and received one-on-one feedback *via* email from the dietitian to promote intervention adherence.

### Outcome assessments

All outcome measures were collected at the University of Kansas Medical Center from December 2018 to February 2020. Demographic information was collected prior to the intervention (baseline) and outcome assessments were completed at baseline and 6 months by trained research assistants. Attendance at group sessions, ovulation, and self-monitoring data were collected weekly *via* self-report using REDCap electronic data capture tools hosted at the University of Kansas Medical Center (35).

#### Ovulation

Each participant was provided both ovulation prediction kits (OPKs; Pregmate, Fort Lauderdale, FL) and OvuSense OvuCore™ fertility sensor (Fertility Focus Inc., Old Saybrook, CT) to predict ovulation occurrence (36). OPKs have a high accuracy (97%) of detecting ovulation, are accessible, non-invasive, and affordable (37) and were used to assess ovulation as the primary outcome. OPKs predict ovulation by detecting the presence of luteinizing hormone (LH) in urine, or the LH surge, which occurs 24–36 h prior to ovulation (38). Participants were instructed to begin urine LH testing on menstrual cycle day 10 and to test every morning until a positive LH surge was seen or until cycle day 18. Participants collected a urine sample each morning upon rising and used the test strip to detect LH.

The OvuSense OvuCore™ fertility sensor provided a second method to detect ovulation through the monitoring of core body temperature. This was used as a secondary device to assess utility and acceptability. This device may help predict ovulation based on the principle that the rise in progesterone in the second half of the cycle results in a rise in basal body temperature (39). The OvuCore™ fertility sensor device measuring 118 mm tip to tail, with a maximum outside diameter of 23 mm was self-inserted into the vagina each night, and recorded core body temperature at 5-min intervals during the night. Once removed upon waking, the sensor was cleaned with soap and water, dried, and connected by the near-field communication (NFC) reader to upload data to the patient's smart phone *via* the OvuSense app. Participants were instructed to start using the sensor the day after menstruation ended and continue using each night until the start of the next menstruation period each month throughout the duration of the study. Ovulation occurrence results from both the OPK's and OvuSense device were self-reported by participants *via* their weekly REDCap survey.

#### Feasibility, attendance, safety, and satisfaction

Retention was measured as the number of women who completed outcome assessment at both baseline and 6 months. Attendance at group sessions was recorded by the dietitian. Group session attendance was defined as being logged in to the video conference and remaining on the screen for the entire 45-min session. Safety was measured as the number of adverse and serious adverse events occurring during the study. Data on intervention acceptance and satisfaction were assessed through an end-of-study survey.

#### Program adherence

Participants tracked their total weekly servings of shakes, entrees, fruits, vegetables, minutes of MVPA, days off plan, weight and steps. Days off plan was defined as eating anything that was not a portion-controlled shake, entrée, fruit, vegetable, non-caloric beverage or allotted 60 kcals of condiments. In addition, weight was recorded by a home scale. These data were self-reported weekly *via* a REDCap survey (35, 40). Weekly totals were reviewed by the dietitian and feedback was provided both individually and in the group meetings to identify barriers and promote social support.

#### Anthropometrics and body composition

Anthropometric and body composition outcomes were collected after a 12-h fast at baseline and month 6. Weight was obtained with participant in a hospital gown by a digital scale (Befour, Inc Model #PS6600, Saukville, WI). Height was measured using a stadiometer. Waist and hip circumference were obtained using the protocol by Lohman et al. (41). Dual-energy x-ray absorptiometry (DXA) was used to measure body composition and specifically fat-free mass, fat mass, and percent body fat (Lunar DPX-IQ, GE Healthcare, Madison, WI). Pregnancy tests were conducted on all women and verified as negative prior to administering the DXA scans. DXA scans were analyzed using native software for Lunar Prodigy Advance (GE Healthcare, Madison, WI). Total fat mass and fat-free mass determined by the sum of lean soft tissue plus bone mineral content were used in the analysis.

#### Dietary intake

Energy and macronutrient intake was measured using self-administered, web-based VioScreen Graphical Food Frequency System (Viocare Technologies, Inc., Princeton, NJ) (42). Additionally, Healthy Eating Index 2010 (HEI-2010) scores were calculated with the data collected in VioScreen. Participants completed the survey at home on a tablet or personal computer within 1 or 2 days following the baseline and 6-month testing visits.

#### Questionnaires

The three-factor eating questionnaire (TFEQ) (43) was used to assess behaviors related to eating, including cognitive restraint of eating, uncontrolled eating or disinhibition, and emotional eating. There are sub-categories within each factor to better understand each of these eating behaviors. Item responses on the TFEQ are scored as 0 or 1 and summed. Higher scores indicate higher levels of restrained eating, disinhibited eating, and predisposition to hunger. PCOS health-related quality of life questionnaire (PCOSQ) (44) was used to assess quality of life and included questions specific to women with PCOS related to body hair, infertility, emotions, menstruation, and weight management.

### Statistics

All statistical tests were 2-tailed and *P* < 0.05 was considered the level of statistical significance. Baseline and demographic characteristics were summarized for the entire sample and completers only (*N* = 8) in Table 1 with continuous variables presented as mean ± standard deviation and categorical variables are presented as percentages and proportions. Primary analyses were conducted on completers only. Time to ovulation was calculated as the difference between the date of self-reported ovulation minus the start date of the intervention. Changes in anthropometrics, body composition, PCOSQ and TFEQ, from baseline to 6 months were assessed *via* Wilcoxon signed-rank tests which were corrected for multiple testing using Hommel's adjustment. Exploratory Wilcoxon signed-rank tests were conducted to assess differences in weight loss between those who met attendance recommendations (attended at least 75% of sessions) and those taking Metformin. Statistical analyses were conducted using SAS 9.4 and R 3.6.3.3.

**Table 1:** Baseline demographics for total sample and completers only.

	**Total**	**Completers**	***P*** **-value**
	***N*** **=** **12**	***N*** **=** **8**	
Age (years)^a^	32.7 (4.2)	32.8 (4.8)	0.87
Non-Hispanic, *n* (%)	4 (33.3)	4 (50)	0.22
Nonwhite, *n* (%)	4 (33.3)	3 (37.5)	0.17
Baseline BMI (kg/m^2^) ^a^	36.8 (4.5)	35.9 (4.2)	0.35
Married, *n* (%)	9 (75)	5 (62.5)	0.37
**Education**			
Some college or more *n*, (%)	11 (92)	7 (87.5)	
Metformin, *n* (%)	5 (42)	4 (50)	0.41

a *Mean (SD)*.

## Results

### Participants/retention/safety

Out of 192 volunteers screened, 14 women met initial eligibility criteria. Primary reasons for exclusion were (1) no longer interested in the study or no response after initial contact (33.7%), (2) outside of BMI range (21.9%), (3) no PCOS diagnosis (18.5%) and 4) prohibitive dietary restrictions (10.1%). Twelve women completed baseline testing and enrolled in the study, with an average age of 33 years, a BMI of 36.8 kg/m^2^, 8 (66%) were white and 11 (92%) were college educated. Eight women (67%) completed 6-month testing. Of the 4 women who did not complete the intervention, 1 became pregnant and 3 did not respond when contacted for 6-month outcome testing. No serious adverse events were reported. There were no baseline differences in those who completed the intervention and those who did not complete the intervention (Table 1).

### Ovulation and menstruation rates

**Figure 1:**
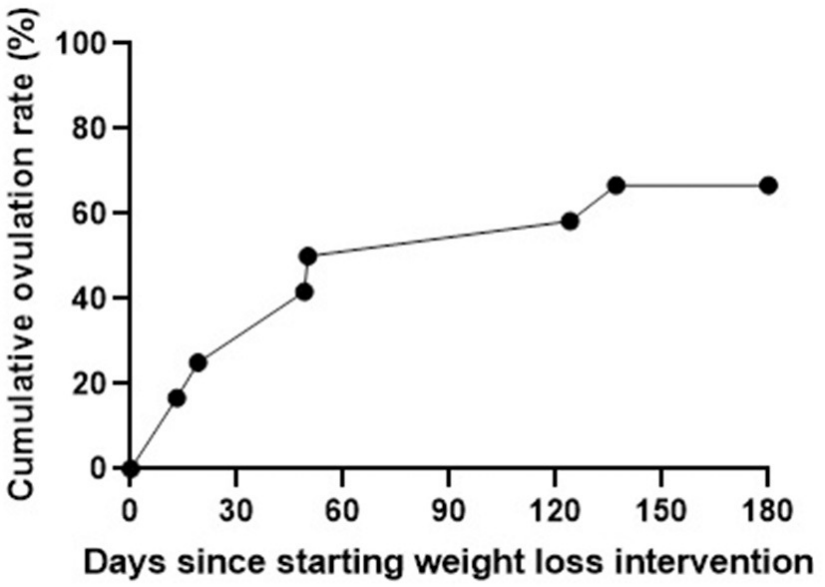
Ovulation rates *via* OPK across weight loss intervention.

On average women reported 2.9 ± 2.0 menstrual cycles during the intervention. Ovulation occurred 1.2 ± 1.2 times as measured by the OPK, and 0.8 ± 0.8 times as measured by the OvuSense OvuCore™ device. Eight (67%) women reported ovulating measured by OPK during the intervention with an average time to ovulation of 57 ± 45 days. Days since starting weight loss intervention and cumulative ovulation rates measured *via* OPK are shown in Figure 1.

### Anthropometrics and body composition

Women lost an average of −3.85 ± 5.94 kg (4.13%, *p* = 0.02), decreased their BMI by 1.61 ± 1.09 kg/m^2^ (*p* = 0.04), and decreased their waist circumference by 4.54 ± 3.03 cm (*p* = 0.04) during the intervention (Table 2). No differences in weight loss were observed between those taking Metformin (*N* = 4; −4.20 ± 2.32%) and those not taking Metformin (*N* = 4; −4.07 ± 3.83%) (*p* = 0.73). The intervention resulted in a loss of fat mass that did not reach statistical significance (−3.33 ± 2.76 kg; *P* = 0.07). There were no significant changes across the intervention for gynoid fat percentage, android fat percentage, body fat percentage, hip circumference or fat-free mass.

**Table 2:** Change in anthropometrics and body composition in study (*n* = 8).

	**Baseline**	**6 month**	**Mean diff**	**SD**	***P*** **-value**
Weight (kg)^*^	95.91	92.06	−3.85	5.94	0.02
% weight loss^*^			−4.13	2.94	0.02
Waist circumference (cm)^*^	101.74	97.21	−4.54	3.03	0.04
% gynoid fat	48.88	48.16	−0.71	1.20	0.29
% android fat	56.89	55.94	−0.95	2.81	0.74
% body fat	47.96	46.24	−1.73	2.75	0.29
Hip circumference (cm)	112.34	110.04	−2.30	7.34	0.51
BMI (kg/m^2^)	35.86	34.25	−1.61	1.09	0.04
Fat mass (kg)	44.64	41.30	−3.33	2.76	0.07
Fat-free mass (kg)	50.19	49.97	−0.22	2.56	0.73
Fat-free mass (kg)	50.19	49.97	−0.22	2.56	0.73

* *p < 0.05*.

### PCOSQ

Self-reported menstrual problems measured by PCOSQ significantly improved over the study (*p* = 0.04) as shown in Table 3. These problems included headaches related to their previous menstrual cycle, irregular menstrual periods, abdominal bloating and menstrual cramps. We did not see any other significant differences across the other domains measured including emotions, body hair, weight and infertility.

**Table 3:** Changes in PCOS related quality of life (*n* = 8).

	**Baseline**	**6 month**	**Mean diff**	***p*** **-value**
Emotions	3.22 ± 0.91	3.70 ± 1.05	0.30 ± 1.01	0.43
Body hair	3.60 ± 1.92	4.53 ± 1.62	0.58 ± 0.72	0.06
Weight	2.09 ± 0.75	3.00 ± 1.56	0.70 ± 1.30	0.17
Infertility problems	2.55 ± 1.95	2.38 ± 1.49	0.06 ± 1.19	0.89
Menstrual problems^*^	2.89 ± 0.93	3.63 ± 0.57	0.47 ± 0.51	0.04

* *p < 0.05*.

### Participant satisfaction

A custom end-of-intervention survey was conducted to measure intervention satisfaction (Table 4). Overall study completers found the intervention to be moderately convenient 4 (50%) or very convenient 4 (50%). All participants were very satisfied 1 (12%) or satisfied 7 (88%) with the intervention. Five (63%) of the participants rated the dietary component slightly difficult, while 3 (37%) rated it moderately difficult. When asked if participants would recommend this approach to weight management to their friends of family, 4 (50%) said yes and the other 4 (50%) reported definitely yes.

**Table 4:** Responses to end study survey (*n* = 8).

**Survey question**	**Response**	**Frequency (%)**
Was this your first time doing a formal weight management program?	Yes	8 (100)
How would you rate the amount of interaction of the other members in your group?	Far too much interaction	0
	Too much interaction	0
	About the right amount	8 (100)
How would you rate the convenience of this intervention?	Moderately convenient	4 (50)
	Very convenient	4 (50)
How satisfied or dissatisfied are you with the intervention?	Very satisfied	1 (13%)
	Satisfied	7 (87%)
How has the intervention met your expectations?	Much better than expected	1 (13)
	Better than I expected	3 (37)
	About what I expected	3 (37)
	Worse than I expected	1 (13)
Do you expect to recommend this approach to a friend interested in weight management?	Yes	4 (50)
	Definitely yes	4 (50)
I am satisfied with the amount of communication I had with my peers.	Strongly agree	1 (13)
	Agree	6 (75)
	Neutral	1 (13)
How difficult was the dietary component of this study?	Slightly difficult	5 (62)
	Moderately difficult	3 (28)
How likely are you to continue using the dietary recommendations outlined in this study?	Somewhat likely	1(13)
	Probably likely	1 (13)
	Certainly likely	6 (75)

### Attendance

On average, attendance at weekly behavioral lessons was 68% (~16 sessions attended out of 24 total). Six (50%) of participants attended the recommended 75% or more of behavioral sessions. Percent weight loss did not differ between those who attended 75% or more behavioral sessions (−4.63 ± 3.28%) compared to who did not (−2.65 ± 0.90%) (*p* = 0.55).

### Three-factor eating

Changes in cognitive restraint of eating, uncontrolled eating or disinhibition, and emotional eating were assessed at baseline and 6-months. We found no differences across the intervention in cognitive restraint of eating (*p* = 0.23), uncontrolled eating (*p* = 0.16), or emotional eating (*p* = 0.09).

### Diet

Valid dietary data was obtained from 4 people at baseline and 6 months. At baseline participants consumed 2.7 servings of fruit/day, 2.9 servings of vegetables/day, had a mean HEI-2010 score of 55.5, and reported energy intake of 1,971 kcals/day. After the 6-month intervention fruit intake increased by ~1 serving per day, vegetable intake increased by ~2.0 servings per day and HEI-2010 scores increased by ~14.5 points per day.

### Adherence to program recommendations

Average weekly totals for entrees, shakes, fruits and vegetables, number of days eating off the meal plan, MVPA and steps are reported in Table 5. Overall, participants did not meet the recommendations for entrees, shakes, and fruits and vegetables; however, they exceeded the recommended MVPA target of 225 min per week with an average of 260 ± 133 min per week during the intervention. The listed amounts of shakes, fruit, vegetable, moderate-vigorous physical activity (MVPA) and steps were the minimum weekly recommendation. Participants were encouraged not to deviate from the allowed foods (off plan) and to attend each week.

**Table 5:** Program recommendations, diet and physical activity adherence and weekly self-monitoring (*n* = 12).

	**Recommendation**	**Mean**	**SD**
Entrees	14/week	13.0	2.9
Shakes	21/week	15.2	5.1
Fruit	17/week	15.3	3.8
Vegetables	18/week	15.8	5.4
Off Plan	0	1.3	0.6
MVPA^a^	225	260	133
Steps	70,000	52,579	15,449
Attendance	75%	68%	21%

a *MVPA, moderate-vigorous physical activity*.

## Discussion

In this study, we investigated the feasibility of a remotely delivered 6-month lifestyle intervention in adult women with PCOS and overweight/obesity and the impact on ovulation rates and time-to-ovulation. First, we observed 66% of the previously anovulatory women in our study resumed ovulation as measured by at least one of our two ovulation detection methods. The average time to ovulation was 57 days with an average self-reported weight loss of −5.3% at time to ovulation. However, we cannot rule out that these women ovulated randomly, and it was independent of the lifestyle intervention. The ovulation rates we observed are lower than a single arm trial by Clark et al. where 81% of the women were anovulatory, 90% of the total sample resumed spontaneous ovulation (16, 45). Our results are more favorable than Mutsaetrs et al. (22) where a diet and exercise intervention prompted resumption of ovulation in 42% of the women perhaps due to a more structured diet and physical activity program.

In our study, women with overweight/obesity and PCOS lost significant weight (4.2%) and decreased their BMI over 6 months following a remotely delivered multicomponent lifestyle intervention. This amount of weight loss is clinically significant and resulted in positive metabolic and psychological outcomes. Our weight loss results at 6 months are similar to Jiskoot et al. in their recently published randomized controlled trial where they found 5.1% weight loss at 12-months in women randomized to a 3-component lifestyle intervention delivered in-person (46).

Additionally, our results for improvement in menstrual cycle symptoms in the menstrual domain of the PCOSQ match results found by Dokras et al. (47) when analyzing the OWL-PCOS study which was a three-arm randomized controlled trial to examine pre-pregnancy interventions including face-to-face lifestyle modification to induce weight loss, hormonal contraception to suppress androgens and the combination of face-to-face lifestyle modification and hormonal contraception on live birth rates in overweight/obese women with PCOS. Additionally, we saw a borderline significant improvement in the body hair domain (*p* = 0.06) of the PCOSQ. Excess body hair (hirsutism) in women with PCOS has been associated with anxiety and depression therefore our lifestyle intervention has the potential to offer psychological benefits as well. Dokras et al. also noted significant improvement in menstrual, weight and fertility domains over a 16-week lifestyle intervention and improvement in all domains (emotion, body hair, menstrual, weight and infertility) in the combined lifestyle intervention and oral contraception pill group (47).

Women with PCOS face many of the same barriers to lifestyle modifications as women without PCOS such as work commitments, time, costs, childcare and access to opportunities for physical activity (48). However, women with PCOS have higher rates of body dissatisfaction, low referral rates to dietitians, more sedentary time per day and consume an additional ~60 kcals/day compared to women without PCOS. Disappointed by the lack of information provided on lifestyle management from the healthcare system, women with PCOS are a notable population that need more resources and innovative options such as remotely delivered lifestyle interventions to improve their health care and quality of life.

There are several strengths of this study, including following the AHA/ACC/TOS guidelines for lifestyle interventions (14). The lifestyle intervention was delivered remotely *via* Skype which allowed participants in a broad geographical region to participate. Body composition was measured using DXA providing insight to the reduction in fat mass across the intervention. We had a very diverse sample (33% minority). PCOS is very heterogeneous, and presentation varies among race and ethnicity therefore it is extremely important to include diverse women in clinical research. End of study surveys noted great acceptance and participant satisfaction. Participants enjoyed the health education component from a registered dietitian and dietary curriculum focused on PCOS.

Limitations of this pilot study include a small sample size and lack of control group due, in part, to stringent eligibility criteria. Many of the patients who initially expressed interest in the study did not qualify because they were on a hormonal form of contraception (oral, intrauterine devices or implant) or declined to participate. In addition, we elected not to include a control group due to the time course nature of fertility. Women must be trying to conceive unsuccessfully for 12 months before being seen and treatment started with a reproductive endocrinologist. For this study, women had to delay treatment further (6 months) for participation in the intervention to test the impact of weight loss on ovulation rates. Therefore, we thought it was unethical to enroll a control group undergoing no fertility treatment or lifestyle intervention for 6 months which is crucial time for this population. Due to the anovulatory nature of this cohort, we were unable to take into account where each participant was at in their current menstrual cycle which led to left censoring/truncation of the data and inability to assess impact of weight loss on time-to-ovulation.

Attrition in this remotely delivered lifestyle intervention was 33%. This level of attrition is similar to lifestyle interventions in the general population (~31%) (49, 50) but lower than lifestyle interventions in women with PCOS that can be anywhere from 50 to 67% (46, 50). Reasons for attrition included becoming pregnant and non-response for the 6-month measurements. Additionally, we noticed that those participants who did not come in for 6-month measurements were not successful with weight loss toward the end of the intervention which is consistent with other reports in the literature. There should be more focus on retention efforts during lifestyle interventions in women with PCOS as those who remain in interventions are more likely to be successful at weight loss.

Implementation of this remotely delivered lifestyle intervention for women with PCOS provides a feasible option for health care providers interested in weight loss for improvement of metabolic, reproductive and psychological features for overweight and obese women with PCOS. Larger and adequately powered trials are necessary to determine the threshold of weight loss necessary induce ovulation and regulate menstruation in overweight and obese women with PCOS.

## Data availability statement

The raw data supporting the conclusions of this article will be made available by the authors, without undue reservation.

## Ethics statement

The studies involving human participants were reviewed and approved by Institutional Review Board of University of Kansas Medical Center. The patients/participants provided their written informed consent to participate in this study.

## Author contributions

AG, LP, CM, and JD: conceptualization. AG and RNM: formal analysis. AG and FS: writing—original draft preparation. AG, LP, FS, CM, JD, RM, RNM, and PS: writing—review and editing. AG: supervision. FS, RM, and PS: project administration. AG and CM: funding acquisition. All authors have read and agreed to the published version of the manuscript. All authors contributed to the article and approved the submitted version.

## Funding

This research was funded by an NIH Clinical and Translational Science Award grant (UL1 TR002366) awarded to the University of Kansas Medical Center (KUMC), and an internal Lied Basic Science Grant Program of the KUMC Research Institute. Additionally, this study was supported by funding from the Department of Obstetrics and Gynecology and Division of Physical Activity and Weight Management at KUMC.

## Conflict of interest

The authors declare that the research was conducted in the absence of any commercial or financial relationships that could be construed as a potential conflict of interest.

## Publisher's note

All claims expressed in this article are solely those of the authors and do not necessarily represent those of their affiliated organizations, or those of the publisher, the editors and the reviewers. Any product that may be evaluated in this article, or claim that may be made by its manufacturer, is not guaranteed or endorsed by the publisher.
